# Distribution of wild bee (Hymenoptera: Anthophila) and hoverfly (Diptera: Syrphidae) communities within farms undergoing ecological transition

**DOI:** 10.3897/BDJ.9.e60665

**Published:** 2021-01-14

**Authors:** Grégoire Noel, Julie Bonnet, Sylvain Everaerts, Anouk Danel, Alix Calderan, Alexis de Liedekerke, Clotilde de Montpellier d'Annevoie, Frédéric Francis, Laurent Serteyn

**Affiliations:** 1 Functional and Evolutionary Entomology, Gembloux Agro-Bio Tech – University of Liège, TERRA, Gembloux, Belgium Functional and Evolutionary Entomology, Gembloux Agro-Bio Tech – University of Liège, TERRA Gembloux Belgium; 2 Ferme de Froidefontaine, Havelange, Belgium Ferme de Froidefontaine Havelange Belgium; 3 Department of Geography, Institute Transitions, University of Namur, Namur, Belgium Department of Geography, Institute Transitions, University of Namur Namur Belgium; 4 Ferme d'Emeville, Havelange, Belgium Ferme d'Emeville Havelange Belgium

**Keywords:** organic and regenerative farming, wild bee, hoverfly, ecological transition

## Abstract

**Background:**

In Havelange (Belgium), two farms are experiencing an ecological transition. We aimed to evaluate the impact of their agricultural activities on insect pollinator communities. This article depicts the situation at the very early stage of the farm transition. This study supports the fact that the maintenance of farm-level natural habitats provides environmental benefits, such as the conservation of two important pollinator communities: wild bees and hoverflies.

**New information:**

Over two years (2018-2019), by using nets and coloured pan-traps, we collected 6301 bee and hoverfly specimens amongst contrasting habitats within two farmsteads undergoing ecological transition in Havelange (Belgium). We reported 101 bee species and morphospecies from 15 genera within six families and 31 hoverfly species and morphospecies from 18 genera. This list reinforces the national pollinator database by providing new distribution data for extinction-threatened species, such as *Andrena schencki* Morawitz 1866, *Bombus campestris* (Panzer 1801), *Eucera longicornis* (L.) and *Halictus maculatus* Smith 1848 or for data deficient species, such as *A. semilaevis* Pérez 1903, *A. fulvata* (Müller 1766), *A. trimmerana* (Kirby 1802) and *Hylaeus brevicornis* Nylander 1852.

## Introduction

Nowadays, the greatest challenge faced by agriculture is to provide food for everyone, without altering the agro-biodiversity and the related ecosystem services (Dendoncker et al. 2018, Duru et al. 2015, Muller et al. 2017). Indeed, the worldwide intensification of agricultural systems has led to tragic biodiversity losses. During the last decades, many studies showed a strong impoverishment of insect pollinators in intensively-farmed landscapes. The depletion of these pollinators - and with them the ecosystem service of pollination - could have severe negative impacts on farmers and consumers welfare (Biesmeijer et al. 2006, Carvalheiro et al. 2013, Potts et al. 2016, Woodcock et al. 2019). The decrease in floral resources and the degradation of nesting sites is one of the main factors of decline (Goulson et al. 2015, Potts et al. 2010, Sánchez-Bayo and Wyckhuys 2019). In Belgium, in 2010, the insect-pollination was valuated at around 250 M€ (Jacquemin et al. 2017).

Agroecological farming systems grow crops on small areas, alongside heterogeneous habitats and complex arrangements (e.g. subdivision of plots by hedgerows, fallow areas, flower meadows etc.) that provide shelters and abundant food resources to beneficial insects (Power et al. 2012). Diversified habitats at the plot or at the farm spatial scale help to control pests, weeds and phytopathogens and provide other regulatory ecosystem services, such as pollination and preservation of nutrients and water in soils (Hatt et al. 2018).

The bee community (Hymenoptera: Anthophila) is amongst the most efficient pollinator groups in temperate agriculture landscapes. In Belgium, the latest inventory recorded 403 bee species, which represents almost one quarter of the European bee diversity (Drossart et al. 2019, Rasmont et al. 2017). Their morphological and behavioural traits co-evolved with flowering plants, allowing them to secure pollination (Michener 2007). The richness of bee morphologies, specialisation in pollen and nectar diets and sizes greatly supports an increase in yields in small-scale agricultural farms (Garibaldi et al. 2016). Since the end of the 19th Century, Belgium has had great expertise in the monitoring of bees. Since the 70s, this survey has particularly accelerated through mapping, preservation and management of historical collections, taxonomic keys and revision of the Belgian fauna (Drossart et al. 2019).

Besides, the Diptera order represents one of the largest and most diverse groups in the pollinator community (Skevington and Dang 2002). Too often neglected, dipteran pollinators ensure the reproduction of many flowering plants (Rader et al. 2015, Ssymank et al. 2008). By consuming pollen and nectar, adult hoverflies (Syrphidae) play a pivotal role in the pollen transmission of over 70% of wildflowers (Doyle et al. 2020, Inouye et al. 2015). Hoverfly larvae exhibit a wide variety of feeding habits, including phytophagy, zoophagy, aphidophagy, saprophagy and mycophagy (Sommaggio 1999). As they cover a large spectrum of microhabitats (e.g. roots layer, herbs layer, dead wood, ponds...) (Speight et al. 2000), hoverfly larvae can be used as biological indicators to evaluate the conservation status of ecosystems (Burgio and Sommaggio 2007, Sommaggio 1999). The widespread distribution of syrphids in temperate landscapes and the availability of excellent taxonomic keys for European species identification are also characteristics that promote syrphids as bio-indicators. Syrphids are very interesting organisms for studying the effects of agriculture intensification on biodiversity because they are particularly mobile (Gao et al. 2020). Moreover, hoverfly communities are strongly affected by the standardisation in landscape structures and by intensive agricultural practices (Dormann et al. 2007). In Belgium, 357 syrphid species were recorded according to the latest survey (Frank Van de Meutter, personal communication).

The impacts of agroecological transition on pollinator communities remain poorly documented. Such evaluation needs standardised and fine-scaled sampling efforts. Thus, the goal of this study is to provide a local and robust inventory of the bee and hoverfly fauna in two farms undergoing ecological transtion in Havelange County (Belgium). The general impacts of farm-scale landscape diversification on bee and hoverfly fauna are discussed. In future research, such inventory will allow an assessment of the impacts of regenerating agricultural landscapes on the pollinator community structure. Moreover, this study feeds in new records and new locations for the national repository of the wild bee and hoverfly communities, owned by the Laboratory of Functional and Evolutionary Entomology (Prof. Frédéric Francis), Gembloux Agro-Bio Tech and the Laboratory of Zoology (Prof. Pierre Rasmont), University of Mons.

## Materials and methods

### Study site and habitats description

**Figure 1.**
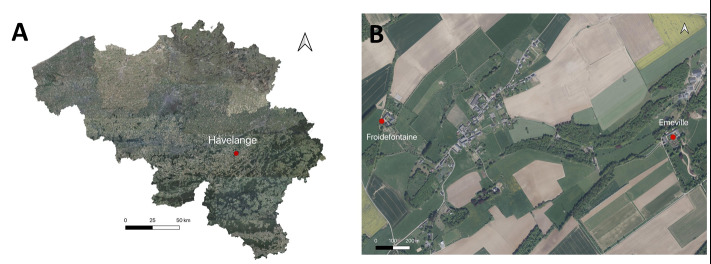
**A.** Location of Havelange Municipality in Belgium; **B.** The location of the two farmsteads in Havelange.

The study was conducted in two neighbouring agricultural sites, located in the Municipality of Havelange (Fig. 1A): the Froidefontaine and Emeville farmsteads (Fig. 1B). They are located at 2 km away from each other, in the geological region of Condroz, in Wallonia (Belgium), as defined by Dufrene and Legendre (1991).

**The Froidefontaine farmstead**

The Froidefontaine farm (50°23'6''N, 5°8'34.799''E) covers an area of 55 hectares, with a mosaic of varied habitats. One of the management objectives is diversifying the land use by conserving natural areas (mesophilic and wet meadows, limestone slopes, ponds...) and hosting different farming projects in a collaborative way on farming areas. Thus, the farm aims at creating a rich and welcoming landscape for diversity, including biodiversity.

**Figure 2.**
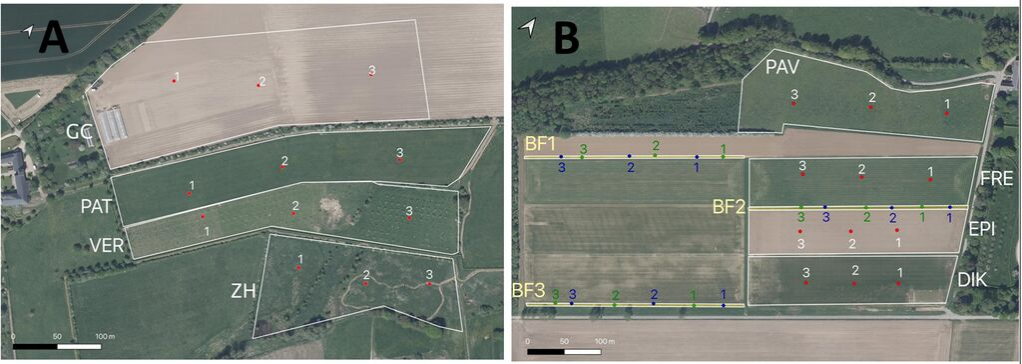
**A.** Froidefontaine farmstead map. GC, PAT, VER and ZH correspond to the sampled parcels, whose details are given in Table 1. Each numbered red dot corresponds to the position of a trio of coloured (white, yellow, blue) pantraps; **B.** Emeville farmstead map. PAV, FRE, EPI and DIK correspond to the sampled parcels, whose details are given in Table 1. Each numbered red dot corresponds to the position of a trio of coloured (white, yellow, blue) pantraps. BF1, BF2 and BF3 correspond to the sampled flower strips. Each blue or green numbered dot corresponds to the position of a trio of coloured (white, yellow, blue) pantraps for the "feeder" flower patch or the "pollinator" flower patch, respectively.

Within the farm, we defined four adjacent habitats (Fig. 2A; Table 1) covering about 10 ha each: a parcel of crops (GC) including a third of the surface with vegetable crops (GC1), a meadow zone (PAT), a young apple orchard (VER) and a wetland (ZH). The parcels were surrounded by hedges principally composed of hornbeam, elderberry, dogwood, hawthorn, maple and European charcoal.

**The Emeville farmstead**

The Emeville farm (50°23'2.4''N 5°10'1.199''E) covers an area of just over 40 ha. In 2016, the farm managers and a committee of various partners converted conventionally-managed fields to agroecological farming methods. To allow a complexification of the ecological network and creating an agricultural landscape enriched with biodiversity, the first actions were: laying hedges and grass strips; planting rustic apple trees; breeding Angus cattle (*Bos taurus taurus* L.) in an orchard; alternating temporary and permanent meadows; arranging of flowered grass strips; using no pesticides and amendments.

The sampling zone covered 15 ha and was divided into seven parcels (Fig. 2B; Table 1), which included six parcels of crops separated by flower strips and one parcel of orchard. Each flower strip (BF1, BF2 and BF3; Fig. 2B) was composed of three plant mix sequences, including a combination of one "feeder" flower patch (BFV) and one "pollinator" flower patch (BFB), separated by the cover crop patch. The cover crop patch was composed of a grass mix of *Festuca arundinacea* Schreb 1771 and *Dactylis glomerata* L. 1753 sown at 20 kg/ha. The feeder flower patch was composed of a mix of 40% of clover (*Trifolium pratense* L. 1753) and 60% of alfalfa (*Medicago sativa* L. 1753) sown at 25 kg/ha. In order to match Agri-Environmental and Climate Measures (AECM) specifications, the pollinator flower patch was sown at 30 kg/ha and was composed of a mix including 85% of grasses (*Poa pratensis* L., 1753 *Festuca rubra* L. 1753 and *Agrostis capillaris* L. 1753), 2% of leguminous species (*Lotus corniculatus* L. 1753, *Medicago lupulina* L. 1753 and *T. pratense*), 3% of annual flower (*Papaver rhoeas* L. 1753, *Glebionis segetum* Fourr. 1869 and *Cyanus segetum* Hill 1762) and 10% of other flower species (*Achillea millefolium* L. 1753, *Centaurea jacea* L. 1753, *Daucus carota* L. 1753, *Leucanthemum vulgare* Lam. 1779, *Malva moschata* L. 1753, *Silene latifolia* Poir. 1789, *Melilotus* sp. Mill. 1754, *Knautia arvensis* Coult. 1828 and *Echium vulgare* L. 1753).

### Collection methods

To assess wild bee and hoverfly diversity, we conducted standardised sampling methods by combining coloured pantraps and netting transects (Földesi and Kovács-Hostyánszki 2014, Westphal et al. 2008, Grundel et al. 2011). Sampling was performed in 2018 and 2019, from April to July. At each collection site (Fig. 2A & B), we positioned a triplet of pantraps (FLORA model with a diameter of 26.5 cm, RINGOT, France) coloured with UV reflecting sprays in white, blue and yellow (ROCOL top tracer model, UK). The pantraps were set-up in line and spaced 3 to 5 metres apart, in order to avoid the attraction coverage bias and to reach the same probabilities of insect capture between the pantraps (Amy et al. 2018, Droege et al. 2010). The pantrap triplets were separated by a minimum of 20 metres, in order to cover each parcel as homogeneously as possible (Carboni and Lebuhn 2003, Eeraerts et al. 2017). Each pantrap was filled with odourless and colourless soapy water every two weeks during one day (from 9:00 AM to 5:00 PM). Every two weeks, we also conducted variable transects with an insect net for one hour in the morning and one hour in the afternoon, for each habitat in Froidefontaine and each flower strip in Emeville (Table 1; Fig. 2). We selected the sampling dates according to the following climatic conditions: temperature higher than 7°C, calm wind (< 12 km/h) and sunny and cloudless day (Westphal et al. 2008). We stocked insects in 70% ethanol for their conservation.

We followed the protocol of Mouret et al. (2007) to prepare, pin and label our collected specimens.

In 2019, we decided to let the yellow pantraps to be continuously activated from mid-May to the end of July with sampling every 10 days to maximise the capture of syrphids and considering that hoverflies have a predilection for the yellow colour (An et al. 2018, Lunau et al. 2018, Wäckers and van Rijn 2012).

### Species identification

Bee specimens were identified at the species level following identification keys of Pauly (2019) for Halictidae, Patiny and Terzo (2010) for Andrenidae and Falk (2015) for the other bee families (Apidae, Colletidae, Megachilidae and Melittidae). All Halicitidae and Andrenidae specimens were confirmed by Alain Pauly (Royal Belgian Institute of Natural Sciences) and Thomas James Wood (University of Mons), respectively. Other bee specimens were confirmed by the reference collections of Gembloux Agro-Bio Tech. Hoverfly specimens were identified at the species level using the identification key of Verlinden (1994). The specimens were then confirmed by Frédéric Francis (University of Liège) and the reference collections of Gembloux Agro-Bio Tech. We applied Belgian Red List of bees for the conservation status of identified species (Drossart et al. 2019).

### Historical data of Havelange Municipality

Thanks to Data Fauna-Flora v.5.1 software (Barbier and Rasmont 2015), we queried the database of Belgian wild bees, on 26 June 2020, for the historical diversity of wild bees in the Havelange Municipality. The selected geographical quadrat was encompassed within latitude from 50°21'14.4''N to 50°24'46.8''N and in longitude from 5°7'12''E to 5°19'26.399''E. The syrphid historical data were not available for Havelange Municipality.

### Statistical analysis

We conducted one-way ANOVA tests to compare species richness and abundance of bee and hoverfly fauna between sampled parcels of Froidefontaine and Emeville farmsteads, separately. We also validated normal distribution of residuals of each ANOVA test. Subsequently, Tukey’s post-hoc tests were used to compare each parcel pair. We separated the flower strips of Emeville farm from the parcel comparisons because they were not sampled with the same effort as those of the sampled parcels. We compared the species richness and abundance of bee and hoverfly fauna between the feeder flower patch (BFV; Fig. 2B) and the pollinator flower patch (BFB; Fig. 2B) using the Student t-test. All statistical analysis were performed using R 4.0.2 (R Development Core Team 2020) and the resulting graphs were built using *ggplot2* and *ggpubr* packages (Kassambara 2020, Wickham 2016).

## Checklists

### Bee Checklist

#### Andrena (Ptilandrena) angustior

(Kirby 1802)

26086597-4FB4-571F-9533-CDBA94D99EB6

##### Ecological interactions

###### Feeds on

Polylectic

###### Conservation status

Near Threatened

##### Notes

Table 2

#### Andrena (Andrena) apicata

Smith 1847

773B4C42-F312-55F1-A798-F2BB489EB06E

##### Ecological interactions

###### Feeds on

Oligolectic on Salicaceae

###### Conservation status

Least Concern

##### Notes

Table 2

#### Andrena (Euandrena) bicolor

Fabricius 1775

AD109654-7ACA-53D3-BDCF-F9CDDEC27AC1

##### Ecological interactions

###### Feeds on

Polylectic

###### Conservation status

Least Concern

##### Notes

Table 2

#### Andrena (Hoplandrena) carantonica

Pérez 1902

87EB1626-D85F-55A6-B7AA-7F30B5637E80

##### Ecological interactions

###### Feeds on

Polylectic

###### Conservation status

Least Concern

##### Notes

Table 2

#### Andrena (Notandrena) chrysosceles

(Kirby 1802)

6E930963-BB1F-514D-9246-C4CD3A7DC3E5

##### Ecological interactions

###### Feeds on

Polylectic

###### Conservation status

Least Concern

##### Notes

Table 2

#### Andrena (Melandrena) cineraria

(Linnaeus 1758)

BB080AAB-19F3-50ED-B714-78A2D50B0F24

##### Ecological interactions

###### Feeds on

Polylectic

###### Conservation status

Least Concern

##### Notes

Table 2

#### Andrena (Andrena) clarkella

(Kirby 1802)

81C96F69-0E63-5D14-8FCE-516BA09F275E

##### Ecological interactions

###### Feeds on

Oligolectic on Salicaceae

###### Conservation status

Least Concern

##### Notes

Table S1 (Historical data)

#### Andrena (Simandrena) dorsata

(Kirby 1802)

ED29E1DB-B7AC-5ADC-9E94-9CDE6778C581

##### Ecological interactions

###### Feeds on

Polylectic

###### Conservation status

Least Concern

##### Notes

Table 2

#### Andrena (Zonandrena) flavipes

Panzer 1799

C4A9DB13-2481-53A0-9C3D-7BB49EB4A3D7

##### Ecological interactions

###### Feeds on

Polylectic

###### Conservation status

Least Concern

##### Notes

Table 2

#### Andrena (Andrena) fulva

(Müller 1776)

2CEB847B-83E8-5DDF-B29C-2CF8B55F2D79

##### Ecological interactions

###### Feeds on

Polylectic

###### Conservation status

Least Concern

##### Notes

Table 2; Table S1 (Historical data)

#### Andrena (Ptilandrena) fulvata

(Müller 1766)

21981822-F1F5-510D-AFE6-D515BBB463E5

##### Ecological interactions

###### Feeds on

Polylectic

###### Conservation status

Non Applicable

##### Notes

Table 2

#### Andrena (Zonandrena) gravida

Imhoff 1832

71CD37F6-AFEF-541A-BE6B-3DF24158A9C7

##### Ecological interactions

###### Feeds on

Polylectic

###### Conservation status

Least Concern

##### Notes

Table 2

#### Andrena (Trachandrena) haemorrhoa

(Fabricius 1781)

3F1DE46C-C113-5073-A9DF-00F991A6FF82

##### Ecological interactions

###### Feeds on

Polylectic

###### Conservation status

Least Concern

##### Notes

Table 2

#### Andrena (Chlorandrena) humilis

Imhoff 1832

4BC96DB5-2547-54FA-AC59-5796792FD9F5

##### Ecological interactions

###### Feeds on

Oligolectic on Asteraceae

###### Conservation status

Least Concern

##### Notes

Table 2

#### Andrena (Holandrena) labialis

(Kirby 1802)

3F6E04EF-30BC-54E6-A83F-0DC065416A8B

##### Ecological interactions

###### Feeds on

Oligolectic on Fabaceae

###### Conservation status

Near Threatened

##### Notes

Table 2

#### Andrena (Poecilandrena) labiata

Fabricius 1781

7FD6F4D7-F0CF-565D-8C12-76B8B4C584B2

##### Ecological interactions

###### Feeds on

Polylectic

###### Conservation status

Least Concern

##### Notes

Table 2

#### Andrena (Micrandrena) minutula

(Kirby 1802)

0757EFDD-4FFA-5AD3-B208-DDDA0C54DD6A

##### Ecological interactions

###### Feeds on

Polylectic

###### Conservation status

Least Concern

##### Notes

Table 2

#### Andrena (Andrena) mitis

Schmiedeknecht 1883

EFA73238-883A-51FA-B945-03D97C2A5D10

##### Ecological interactions

###### Feeds on

Oligolectic on Salicaceae

###### Conservation status

Least Concern

##### Notes

Table 2

#### Andrena (Melandrena) nigroaenea

(Kirby 1802)

381F5A71-18CB-54ED-80A9-03EEF9D0F6B6

##### Ecological interactions

###### Feeds on

Polylectic

###### Conservation status

Least Concern

##### Notes

Table 2

#### Andrena (Melandrena) nitida

(Müller 1776)

B7C2E8AB-ECC3-5B86-82A0-D23A612F88C1

##### Ecological interactions

###### Feeds on

Polylectic

###### Conservation status

Least Concern

##### Notes

Table 2

#### Andrena (Taeniandrena) ovatula

(Kirby 1802)

9605C2AF-A8A2-5C8B-9065-8140A0BD9C3F

##### Ecological interactions

###### Feeds on

Polylectic

###### Conservation status

Near Threatened

##### Notes

Table 2

#### Andrena (Andrena) praecox

(Scopoli 1763)

623D6A91-EABB-51EF-AA08-B0CF915AB796

##### Ecological interactions

###### Feeds on

Oligolectic on Salicaceae

###### Conservation status

Least Concern

##### Notes

Table 2

#### Andrena (Opandrena) schencki

Morawitz 1866

ADB6FEBD-15C2-53D5-911A-882297C0CBA2

##### Ecological interactions

###### Feeds on

Polylectic

###### Conservation status

Endangered

##### Notes

Table 2

#### Andrena (Micrandrena) semilaevis

Pérez 1903

894446EE-D3EC-5DC0-B6DB-607AAAF27820

##### Ecological interactions

###### Feeds on

Polylectic

###### Conservation status

Data Deficient

##### Notes

Table 2

#### Andrena (Micrandrena) subopaca

Nylander 1848

05B5B4F5-9688-5D29-B9D4-A4851FD3ED1B

##### Ecological interactions

###### Feeds on

Polylectic

###### Conservation status

Least Concern

##### Notes

Table 2

#### Andrena (Hoplandrena) trimmerana

(Kirby 1802)

5CBA98DF-9E49-577B-9EB7-5F0D6571780C

##### Ecological interactions

###### Feeds on

Polylectic

###### Conservation status

Data Deficient

##### Notes

Table 2

#### Andrena (Melandrena) vaga

Panzer 1799

0167CEF8-A916-5A3E-92F9-50DFACF19429

##### Ecological interactions

###### Feeds on

Oligolectic on Salicaceae

###### Conservation status

Least Concern

##### Notes

Table 2

#### Andrena (Taeniandrena) wilkella

(Kirby 1802)

0887060D-D9C4-5E04-8D23-A300E971654F

##### Ecological interactions

###### Feeds on

Polylectic

###### Conservation status

Near Threatened

##### Notes

Table 2

#### Anthophora plumipes

(Pallas 1772)

AC896C77-7C6F-5B5F-9E52-93ABED2376D4

##### Ecological interactions

###### Feeds on

Polylectic

###### Conservation status

Least Concern

##### Notes

Table S1 (Historical data)

#### Apis mellifera

Linnaeus 1758

05A46C7F-94FE-5EC9-9656-C7D8040B79B1

##### Ecological interactions

###### Feeds on

Polylectic

###### Conservation status

Data Deficient

##### Notes

Table 2

#### Bombus (Ashtonipsithyrus) bohemicus

Seidl 1837

E9BB06B7-2300-5E66-8602-C71D1EBFCDB0

##### Ecological interactions

###### Feeds on

Cuckoo bee

###### Conservation status

Near Threatened

##### Notes

Table S1 (Historical data)

#### Bombus (Psithyrus) campestris

(Panzer 1801)

25C5E9BD-094C-5215-BE6C-BBDD2CFF0872

##### Ecological interactions

###### Feeds on

Cuckoo bee

###### Conservation status

Vulnerable

##### Notes

Table 2

#### Bombus (Bombus) cryptarum

(Fabricius 1775)

A15CB08A-5268-5E21-A817-536C1D11A458

##### Ecological interactions

###### Feeds on

Polylectic

###### Conservation status

Endangered

##### Notes

Table S1 (Historical data)

#### Bombus (Megabombus) hortorum

(Linnaeus 1761)

EC9C9D09-E1D7-5784-990F-B7A98849516C

##### Ecological interactions

###### Feeds on

Polylectic

###### Conservation status

Near Threatened

##### Notes

Table 2; Table S1 (Historical data)

#### Bombus (Pyrobombus) hypnorum

(Linnaeus 1758)

FC891598-8EAA-5AE9-8E39-07F7811A92A1

##### Ecological interactions

###### Feeds on

Polylectic

###### Conservation status

Least Concern

##### Notes

Table 2

#### Bombus (Melanobombus) lapidarius

(Linnaeus 1758)

FD78CC14-D995-5950-9500-12B22441EC65

##### Ecological interactions

###### Feeds on

Polylectic

###### Conservation status

Least Concern

##### Notes

Table 2; Table S1 (Historical data)

#### Bombus (Bombus) lucorum

(Linnaeus 1761)

ED1342B1-7762-5A13-B217-22BE78486F58

##### Ecological interactions

###### Feeds on

Polylectic

###### Conservation status

Near Threatened

##### Notes

Table S1 (Historical data)

#### Bombus (Thoracobombus) pascuorum

(Scopoli 1793)

DF277625-98E5-53C1-83DD-FE023E20062E

##### Ecological interactions

###### Feeds on

Polylectic

###### Conservation status

Least Concern

##### Notes

Table 2; Table S1 (Historical data)

#### Bombus (Pyrobombus) pratorum

(Linnaeus 1761)

D29BB35F-A195-5EDD-8913-650982CC35CE

##### Ecological interactions

###### Feeds on

Polylectic

###### Conservation status

Least Concern

##### Notes

Table 2; Table S1 (Historical data)

#### Bombus (Thoracobombus) ruderarius

(Müller, 1776)

FE4DF810-ED84-592D-8885-9EA17CB932BA

##### Ecological interactions

###### Feeds on

Polylectic

###### Conservation status

Endangered

##### Notes

Table S1 (Historical data)

#### Bombus (Fernaldaepsithyrus) sylvestris

(Lepeletier 1832)

A6010E96-5C5C-51A8-9ABC-797EA5E12485

##### Ecological interactions

###### Feeds on

Cuckoo bee

###### Conservation status

Least Concern

##### Notes

Table S1 (Historical data)

#### Bombus (Bombus) terrestris

(Linnaeus 1758)

BB1DFE9F-B620-52EB-B70F-87252F86243F

##### Ecological interactions

###### Feeds on

Polylectic

###### Conservation status

Least Concern

##### Notes

Table 2; Table S1 (Historical data)

#### Bombus (Psithyrus) vestalis

(Fourcroy 1785)

1E6E660B-A243-58B7-A5CC-6D2EFA69BB3D

##### Ecological interactions

###### Feeds on

Cuckoo bee

###### Conservation status

Near Threatened

##### Notes

Table 2

#### Chelostoma rapunculi

(Lepeletier 1841)

38ECE448-31C1-5881-ABFB-21B4F172A1EE

##### Ecological interactions

###### Feeds on

Oligolectic on Campanulaceae

###### Conservation status

Least Concern

##### Notes

Table 2

#### Colletes cunicularius

(Linnaeus 1758)

705C93B0-B361-57A5-AA37-7BCCD4669F94

##### Ecological interactions

###### Feeds on

Polylectic

###### Conservation status

Least Concern

##### Notes

Table 2

#### Colletes daviesanus

Smith 1846

70F62270-5245-5F6B-A23B-F94E9500E9F8

##### Ecological interactions

###### Feeds on

Oligolectic on Asteraceae

###### Conservation status

Least Concern

##### Notes

Table 2

#### Eucera (Eucera) longicornis

(Linnaeus 1758)

598FC1FF-9AAF-5F2E-AF73-6BEECF404049

##### Ecological interactions

###### Feeds on

Oligolectic on Orchidaceae

###### Conservation status

Vulnerable

##### Notes

Table 2

#### Halictus maculatus

Smith 1848

31725763-5194-5955-9C1C-F38B6D1B720F

##### Ecological interactions

###### Feeds on

Polylectic

###### Conservation status

Vulnerable

##### Notes

Table 2

#### Halictus rubicundus

(Christ 1791)

873F7A0D-478D-5319-B02F-06192CEE40E6

##### Ecological interactions

###### Feeds on

Polylectic

###### Conservation status

Least Concern

##### Notes

Table 2

#### Halictus scabiosae

(Rossi 1790)

9805CF81-2787-50D2-BC4B-13A5A9BD6A5F

##### Ecological interactions

###### Feeds on

Polylectic

###### Conservation status

Least Concern

##### Notes

Table 2

#### Hylaeus brevicornis

Nylander 1852

AD123417-6224-5AD5-9C57-C000FE474C9A

##### Ecological interactions

###### Feeds on

Polylectic

###### Conservation status

Data Deficient

##### Notes

Table 2

#### Hylaeus communis

Nylander 1852

DCF6AA3F-43FC-5D8E-A687-281AE90D93A0

##### Ecological interactions

###### Feeds on

Polylectic

###### Conservation status

Least Concern

##### Notes

Table 2

#### Hylaeus hyalinatus

Smith 1842

90D4646E-40CB-5970-B767-EF525A6E7DA7

##### Ecological interactions

###### Feeds on

Polylectic

###### Conservation status

Least Concern

##### Notes

Table 2

#### Hylaeus signatus

(Panzer 1798)

17DA14F9-6F8B-55C0-B28B-4D4AA5AFCD21

##### Ecological interactions

###### Feeds on

Oligolectic on Resedaceae

###### Conservation status

Least Concern

##### Notes

Table 2

#### Lasioglossum (Evylaeus) calceatum

(Scopoli 1763)

1744D1E1-A2EA-56FA-9526-6D06CD3CB790

##### Ecological interactions

###### Feeds on

Polylectic

###### Conservation status

Least Concern

##### Notes

Table 2

#### Lasioglossum (Evylaeus) fulvicorne

(Kirby 1802)

B118C370-2825-5A69-82BF-6567D8A808BD

##### Ecological interactions

###### Feeds on

Polylectic

###### Conservation status

Least Concern

##### Notes

Table 2

#### Lasioglossum (Evylaeus) laticeps

(Schenck 1868)

A7DEA072-A4D9-5DB8-9857-4AA2DE5A6BBE

##### Ecological interactions

###### Feeds on

Polylectic

###### Conservation status

Least Concern

##### Notes

Table 2

#### Lasioglossum (Lasioglossum) lativentre

(Schenck 1853)

A88DF8F3-10EE-56C5-9AA6-65F265BA0C45

##### Ecological interactions

###### Feeds on

Polylectic

###### Conservation status

Least Concern

##### Notes

Table 2

#### Lasioglossum (Evylaeus) leucopus

(Kirby 1802)

2573CE7F-7651-5B23-8AE5-FF375F6453CE

##### Ecological interactions

###### Feeds on

Polylectic

###### Conservation status

Near Threatened

##### Notes

Table 2

#### Lasioglossum (Lasioglossum) leucozonium

(Schrank 1781)

3DC5D884-DD48-5E14-9D6C-BA11B18FB7AD

##### Ecological interactions

###### Feeds on

Polylectic

###### Conservation status

Least Concern

##### Notes

Table 2

#### Lasioglossum (Evylaeus) malachurum

(Kirby 1802)

5417ECA7-9629-5D8F-8E10-DEE75FC37A81

##### Ecological interactions

###### Feeds on

Polylectic

###### Conservation status

Least Concern

##### Notes

Table 2

#### Lasioglossum (Evylaeus) morio

(Fabricius 1793)

16749B48-4DA5-5028-97BE-C1EB97D7E223

##### Ecological interactions

###### Feeds on

Polylectic

###### Conservation status

Least Concern

##### Notes

Table 2

#### Lasioglossum (Evylaeus) pauxillum

(Schenck 1853)

603F3E27-208A-5D25-853F-8399862F1ECE

##### Ecological interactions

###### Feeds on

Polylectic

###### Conservation status

Least Concern

##### Notes

Table 2

#### Lasioglossum (Evylaeus) punctatissimum

(Schenck 1853)

B3B32BA7-8805-5918-8FB7-4FDA39BD2C04

##### Ecological interactions

###### Feeds on

Polylectic

###### Conservation status

Least Concern

##### Notes

Table 2

#### Lasioglossum (Evylaeus) sexstrigatum

(Schenck 1868)

226CB23B-196D-54F9-B303-CA041622F181

##### Ecological interactions

###### Feeds on

Polylectic

###### Conservation status

Least Concern

##### Notes

Table 2

#### Lasioglossum sp.

3EF7CA5D-A3E8-565A-B100-41D44F98D254

##### Notes

Table 2

#### Lasioglossum (Evylaeus) villosulum

(Kirby 1802)

6249CCA0-08DF-5719-8C8F-2B8E01DA95D6

##### Ecological interactions

###### Feeds on

Polylectic

###### Conservation status

Least Concern

##### Notes

Table 2

#### Lasioglossum (Lasioglossum) zonulum

(Smith 1848)

1D1588AE-FEE5-57F5-9A3B-B6FE878400B8

##### Ecological interactions

###### Feeds on

Polylectic

###### Conservation status

Least Concern

##### Notes

Table 2

#### Megachile ericetorum

Lepeletier 1841

70278B89-1686-5385-ADEA-6CC72A9FF609

##### Ecological interactions

###### Feeds on

Oligolectic on Fabaceae

###### Conservation status

Least Concern

##### Notes

Table 2

#### Megachile willughbiella

(Kirby 1802)

D129DEFE-0678-5879-B31C-7EB786CA440E

##### Ecological interactions

###### Feeds on

Polylectic

###### Conservation status

Least Concern

##### Notes

Table 2

#### Melitta leporina

(Panzer 1799)

C97B8D1F-79F4-59D6-91D2-B19766E7ADC3

##### Ecological interactions

###### Feeds on

Oligolectic on Orobanchaceae

###### Conservation status

Least Concern

##### Notes

Table 2

#### Nomada bifasciata

Olivier 1811

9B1667C8-7517-5CB2-98D0-AF1BAF0B0110

##### Ecological interactions

###### Feeds on

Cuckoo bee

###### Conservation status

Least Concern

##### Notes

Table 2

#### Nomada fabriciana

Panzer 1798

B7E531D8-4E55-501A-8291-00D011328259

##### Ecological interactions

###### Feeds on

Cuckoo bee

###### Conservation status

Least Concern

##### Notes

Table 2

#### Nomada facilis

Schwarz 1967

D8FC3639-B920-55DD-8C1F-FB7EDDC97407

##### Ecological interactions

###### Feeds on

Cuckoo bee

###### Conservation status

Least Concern

##### Notes

Table S1 (Historical data)

#### Nomada flava

(Kirby 1802)

FDD19373-C8B3-594E-AEAE-72C723F678A3

##### Ecological interactions

###### Feeds on

Cuckoo bee

###### Conservation status

Least Concern

##### Notes

Table 2; Table S1 (Historical data)

#### Nomada flavoguttata

Panzer 1798

34B6F864-C91E-5EB0-AF52-646D83B27C25

##### Ecological interactions

###### Feeds on

Cuckoo bee

###### Conservation status

Least Concern

##### Notes

Table 2

#### Nomada fucata

Fabricius 1793

D4427144-9927-5D2B-82B1-A398030DA9EA

##### Ecological interactions

###### Feeds on

Cuckoo bee

###### Conservation status

Least Concern

##### Notes

Table 2

#### Nomada fulvicornis

(Kirby 1802)

39786D32-B140-5908-841B-7F266AB5A941

##### Ecological interactions

###### Feeds on

Cuckoo bee

###### Conservation status

Least Concern

##### Notes

Table 2

#### Nomada goodeniana

(Kirby 1802)

93F8F825-D4D8-56F7-AC2B-3E8B9CC82373

##### Ecological interactions

###### Feeds on

Cuckoo bee

###### Conservation status

Least Concern

##### Notes

Table 2

#### Nomada integra

Brulé 1832

2C19D6AD-D14C-5E5C-A527-AF76683E2564

##### Ecological interactions

###### Feeds on

Cuckoo bee

###### Conservation status

Vulnerable

##### Notes

Table S1 (Historical data)

#### Nomada lathburiana

(Kirby 1802)

9F67A09B-2019-5932-AA8C-95F007FD9DE5

##### Ecological interactions

###### Feeds on

Cuckoo bee

###### Conservation status

Least Concern

##### Notes

Table 2

#### Nomada leucophthalma

(Kirby 1802)

92AC6EA0-A3BA-51E4-BC86-D60C5CEC225F

##### Ecological interactions

###### Feeds on

Cuckoo bee

###### Conservation status

Least Concern

##### Notes

Table 2

#### Nomada marshamella

Lepeletier 1841

55CC1179-8DCE-5C1D-A06E-7A4FD614D928

##### Ecological interactions

###### Feeds on

Cuckoo bee

###### Conservation status

Least Concern

##### Notes

Table 2; Table S1 (Historical data)

#### Nomada panzeri

(Linnaeus 1758)

D8D544B6-87EC-5E22-8623-0FED8C8162FF

##### Ecological interactions

###### Feeds on

Cuckoo bee

###### Conservation status

Least Concern

##### Notes

Table 2

#### Nomada ruficornis

Fabricius 1793

06000374-DEBA-546D-844E-71E384D5E8FE

##### Ecological interactions

###### Feeds on

Cuckoo bee

###### Conservation status

Least Concern

##### Notes

Table 2; Table S1 (Historical data)

#### Nomada signata

Jurine 1807

6BF255D1-42E2-562A-A60F-A1DC7B0CBA32

##### Ecological interactions

###### Feeds on

Cuckoo bee

###### Conservation status

Least Concern

##### Notes

Table 2

#### Nomada succincta

Panzer 1798

D6C94E98-8489-5156-B17C-77F5CD03AA46

##### Ecological interactions

###### Feeds on

Cuckoo bee

###### Conservation status

Least Concern

##### Notes

Table 2

#### Nomada zonata

Panzer 1798

60709FC6-A497-5FE3-A8C0-E50BC33FF16B

##### Ecological interactions

###### Feeds on

Cuckoo bee

###### Conservation status

Least Concern

##### Notes

Table 2

#### Osmia bicolor

(Schrank 1781)

948C4CAF-20AC-5B9F-8FFD-0E90A5FBEA2A

##### Ecological interactions

###### Feeds on

Polylectic

###### Conservation status

Least Concern

##### Notes

Table 2

#### Osmia bicornis

(Linnaeus 1758)

491182D3-2281-5F9A-8B14-5CB8E9B4720A

##### Ecological interactions

###### Feeds on

Polylectic

###### Conservation status

Least Concern

##### Notes

Table 2

#### Osmia cornuta

(Latreille 1805)

560596E3-1F94-50A9-B1E4-A9054049D5D8

##### Ecological interactions

###### Feeds on

Polylectic

###### Conservation status

Least Concern

##### Notes

Table 2

#### Osmia leaiana

(Kirby 1802)

CABA8176-0B1E-55F3-9B7B-72D3C19042B4

##### Ecological interactions

###### Feeds on

Oligolectic on Asteraceae

###### Conservation status

Least Concern

##### Notes

Table 2

#### Osmia leucomelana

(Kirby 1802)

23711571-D04D-5663-AB09-638C6B483ADB

##### Ecological interactions

###### Feeds on

Polylectic

###### Conservation status

Least Concern

##### Notes

Table 2

#### Osmia tridentata

Dufour & Perris 1840

D33C3091-21CA-5E49-B160-C814AC38702D

##### Ecological interactions

###### Feeds on

Polylectic

###### Conservation status

Least Concern

##### Notes

Table 2

#### Seladonia tumulorum

(Linnaeus 1758)

64AB9D91-37AB-5194-83D8-5A4311AA2ADF

##### Ecological interactions

###### Feeds on

Polylectic

###### Conservation status

Least Concern

##### Notes

Table 2

#### Sphecodes ephippius

(Linnaeus 1767)

7F29730F-2965-586C-BFC1-D8CADDB49A3E

##### Ecological interactions

###### Feeds on

Cuckoo bee

###### Conservation status

Least Concern

##### Notes

Table 2

#### Sphecodes ferruginatus

Hagens 1882

5CD3E34E-6546-5806-BBD7-4EDD0A124711

##### Ecological interactions

###### Feeds on

Cuckoo bee

###### Conservation status

Least Concern

##### Notes

Table 2

#### Sphecodes gibbus

(Linnaeus 1758)

8FC2E3A1-A810-5BC0-BACA-D5C7D2D0E5BD

##### Ecological interactions

###### Feeds on

Cuckoo bee

###### Conservation status

Least Concern

##### Notes

Table 2

#### Sphecodes monilicornis

(Kirby 1802)

76A571DE-6A83-59DA-8058-E74BC4176C49

##### Ecological interactions

###### Feeds on

Cuckoo bee

###### Conservation status

Least Concern

##### Notes

Table 2

#### Sphecodes puncticeps

Thomson 1870

E9CF53B2-A974-51ED-99DD-168D066F0762

##### Ecological interactions

###### Feeds on

Cuckoo bee

###### Conservation status

Least Concern

##### Notes

Table 2

#### Sphecodes sp.

5C8E9C69-5C10-5A06-9F9F-F0600257FC2B

##### Ecological interactions

###### Feeds on

Cuckoo bee

##### Notes

Table 2

### Hoverfly Checklist

#### Cheilosia sp.

Meigen 1822

90050123-9179-5E3F-8E07-2EDAA3F9F3AF

##### Ecological interactions

###### Feeds on

Polylectic

###### Conservation status

Not Applicable

##### Notes

Table 2

#### Episyrphus balteatus

(De Geer 1776)

53CF093A-375A-5B54-AD1B-B8F688A04037

##### Ecological interactions

###### Feeds on

Polylectic

###### Conservation status

Not Applicable

##### Notes

Table 2

#### Eristalis arbustorum

(Linnaeus 1758)

F5FEA349-0334-5849-8ADC-53CC3A9818C9

##### Ecological interactions

###### Feeds on

Polylectic

###### Conservation status

Not Applicable

##### Notes

Table 2

#### Eristalis nemorum

(Linnaeus 1758)

BA3E4C59-DBF8-5529-8093-97F86233CFD4

##### Ecological interactions

###### Feeds on

Polylectic

###### Conservation status

Not Applicable

##### Notes

Table 2

#### Eristalis pertinax

(Scopoli 1763)

BB83C0FB-CBEE-5FD3-9F69-410762CC14A9

##### Ecological interactions

###### Feeds on

Polylectic

###### Conservation status

Not Applicable

##### Notes

Table 2

#### Eristalis sepulchralis

(Linnaeus 1758)

93FF6A50-A0D4-5117-B746-91EE0DD66B8C

##### Ecological interactions

###### Feeds on

Polylectic

###### Conservation status

Not Applicable

##### Notes

Table 2

#### Eristalis similis

(Fallèn 1817)

F89CB51F-CF0A-5D74-9E32-19E1A7A2B421

##### Ecological interactions

###### Feeds on

Polylectic

###### Conservation status

Not Applicable

##### Notes

Table 2

#### Eristalis tenax

(Linnaeus 1758)

38B88F15-9565-5863-A262-EAA792C67AC3

##### Ecological interactions

###### Feeds on

Polylectic

###### Conservation status

Not Applicable

##### Notes

Table 2

#### Eupeodes luniger

(Meigen 1822)

C838CF26-339D-59ED-8A20-93B7FB48C12C

##### Ecological interactions

###### Feeds on

Polylectic

###### Conservation status

Not Applicable

##### Notes

Table 2

#### Ferdinandea cuprea

(Scopoli 1763)

7D8EA026-B075-5EB9-90B5-FE9A80268201

##### Ecological interactions

###### Feeds on

Polylectic

###### Conservation status

Not Applicable

##### Notes

Table 2

#### Helophilus trivittatus

(Fabricius 1805)

EDEACC39-7F2D-53BB-AFA9-4C3ABC0D3CE0

##### Ecological interactions

###### Feeds on

Polylectic

###### Conservation status

Not Applicable

##### Notes

Table 2

#### Melanostoma mellinum

(Linnaeus 1758)

481349F2-DD00-5900-ABDE-5D750AA7A354

##### Ecological interactions

###### Feeds on

Polylectic

###### Conservation status

Not Applicable

##### Notes

Table 2

#### Metasyrphus corollae

(Fabricius 1794)

BF63ABD8-86F0-5ABB-B60D-62979A7E15CB

##### Ecological interactions

###### Feeds on

Polylectic

###### Conservation status

Not Applicable

##### Notes

Table 2

#### Metasyrphus latifasciatus

(Macquart 1829)

61023682-D938-50BD-8BB7-E2E6FC8A5E51

##### Ecological interactions

###### Feeds on

Polylectic

###### Conservation status

Not Applicable

##### Notes

Table 2

#### Myathropa florea

(Linnaeus 1758)

B251790A-E0FA-5E46-935D-612EB37AB9F5

##### Ecological interactions

###### Feeds on

Polylectic

###### Conservation status

Not Applicable

##### Notes

Table 2

#### Platycheirus albimanus

(Fabricius 1781)

290814F9-DC8A-5E26-BA25-ADDCB4733474

##### Ecological interactions

###### Feeds on

Polylectic

###### Conservation status

Not Applicable

##### Notes

Table 2

#### Platycheirus clypeatus

(Meigen 1822)

7F670446-E654-5BA3-9D0D-1EBA9F50B8EB

##### Ecological interactions

###### Feeds on

Polylectic

###### Conservation status

Not Applicable

##### Notes

Table 2

#### Platycheirus immarginatus

(Zetterstedt 1849)

7B152F87-B0F6-562C-A25E-35A4A4C4E6FC

##### Ecological interactions

###### Feeds on

Oligolectic on Cyperaceae

###### Conservation status

Not Applicable

##### Notes

Table 2

#### Platycheirus peltatus

(Meigen 1822)

B7F1B06F-01ED-52C0-970D-4F85A42CF4B1

##### Ecological interactions

###### Feeds on

Polylectic

###### Conservation status

Not Applicable

##### Notes

Table 2

#### Platycheirus scambus

(Staeger 1843)

4F178E3D-398F-5E6F-8C92-D1282CA7E6E5

##### Ecological interactions

###### Feeds on

Polylectic

###### Conservation status

Not Applicable

##### Notes

Table 2

#### Rhingia campestris

Meigen 1822

B0EFBFC0-2AC5-515D-B792-F22104462E47

##### Ecological interactions

###### Feeds on

Polylectic

###### Conservation status

Not Applicable

##### Notes

Table 2

#### Scaeva pyrastri

(Linnaeus 1758)

DF7175F0-5E96-5F27-BEF1-7E2AD03539C2

##### Ecological interactions

###### Feeds on

Polylectic

###### Conservation status

Not Applicable

##### Notes

Table 2

#### Sphaerophoria scripta

(Linnaeus 1758)

2B6BC5BE-26CA-50ED-A3CB-0A6837186BBF

##### Ecological interactions

###### Feeds on

Polylectic

###### Conservation status

Not Applicable

##### Notes

Table 2

#### Syritta pipiens

(Linnaeus 1758)

CD7067BB-457C-5FB1-BDFA-12FC949916D4

##### Ecological interactions

###### Feeds on

Polylectic

###### Conservation status

Not Applicable

##### Notes

Table 2

#### Syrphus ribesii

(Linnaeus 1758)

50F55B3F-2078-5B9D-877E-0576C6AA7543

##### Ecological interactions

###### Feeds on

Polylectic

###### Conservation status

Not Applicable

##### Notes

Table 2

#### Syrphus vitripennis

Meigen 1822

EEBD48C0-87E0-5828-99B5-DB19EE78F53D

##### Ecological interactions

###### Feeds on

Polylectic

###### Conservation status

Not Applicable

##### Notes

Table 2

#### Volucella bombylans

(Linnaeus 1758)

A3F2FF42-4B36-58EA-84A0-9CA20C7B2EB1

##### Ecological interactions

###### Feeds on

Polylectic

###### Conservation status

Not Applicable

##### Notes

Table 2

#### Volucella pellucens

(Linnaeus 1758)

3296E90F-34DA-550D-8AC4-22300A23FE44

##### Ecological interactions

###### Feeds on

Polylectic

###### Conservation status

Not Applicable

##### Notes

Table 2

#### Xanthogramma pedissequum

(Harris 1776)

226D97AC-74A4-570F-B298-AFCF80C8226F

##### Ecological interactions

###### Feeds on

Polylectic

###### Conservation status

Not Applicable

##### Notes

Table 2

#### Xylota segnis

(Linnaeus 1758)

F9132A36-B891-5F8F-B53F-492985E4EC15

##### Ecological interactions

###### Feeds on

Polylectic

###### Conservation status

Not Applicable

##### Notes

Table 2

#### Xylota sylvarum

(Linnaeus 1758)

9D5FAD45-007C-5431-B5CD-E6176EFA5110

##### Ecological interactions

###### Feeds on

Polylectic

###### Conservation status

Not Applicable

##### Notes

Table 2

## Analysis

### Collection results

Over 2 years (2018-2019) of sampling, we collected 4,303 bees and 1,998 syrphids, representing 92 species and morphospecies from 15 genera and six families for the bees and 31 species and morphospecies from 18 genera for the hoverflies (Table 2). Polylectic, oligolectic and cuckoo bee species correspond to 61%, 14% and 25% of bee richness, respectively. However, the relative proportion of specialised bee (0.9%) was low, with polylectic and cuckoo bees corresponding to 94% and 5.1% in abundance of the total sampled bees, respectively (Table 2). All adult hoverfly species were considered as polylectic species (Frank Van de Meutter, pers. comm.), except for *Platycheirus immarginatus* (Table 2). In the historical database of Belgian wild bees, we retrieved 18 bee species corresponding to 349 specimens between 1918 and 2007. These data are available in Suppl. material 1. With these historical data of the Havelange Municipality, the bee diversity reached 101 different bee species.

### Statistical analysis

**Figure 3.**
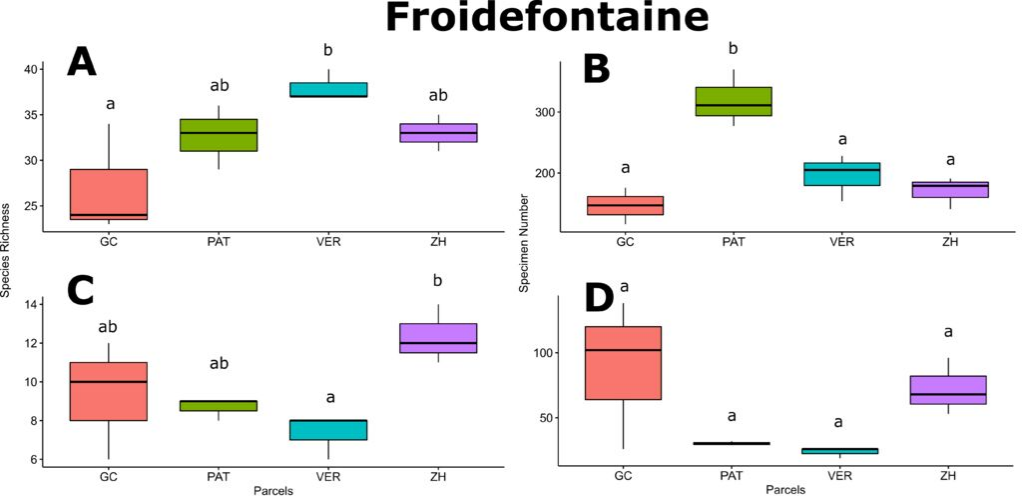
Mean values of species richness and abundance for bee and hoverfly fauna amongst Froidefontaine parcels GC, PAT, VER and ZH (see details given in Table 1). **A.** Bee richness; **B.** Bee abundance; **C.** Hoverfly richness; **D.** Hoverfly abundance. Letters above the boxplots represent Tukey's post-hoc comparisons.

**Figure 4.**
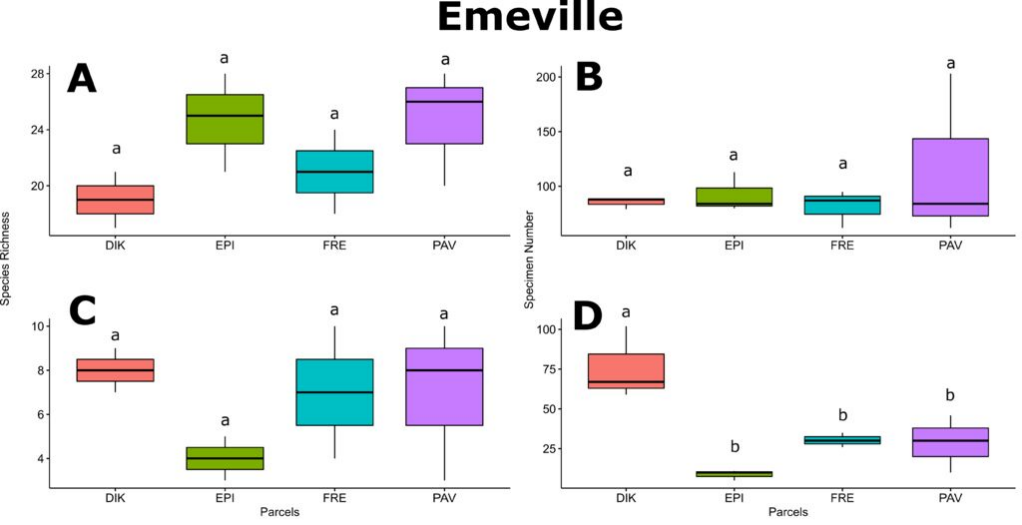
Mean values of species richness and abundance for bee and hoverfly fauna amongst Emeville parcels DIK, EPI, FRE and PAV (see details given in Table 1). **A.** Bee richness; **B.** Bee abundance; **C.** Hoverfly richness; **D.** Hoverfly abundance. Letters above the boxplots represent Tukey's post-hoc comparisons.

**Figure 5.**
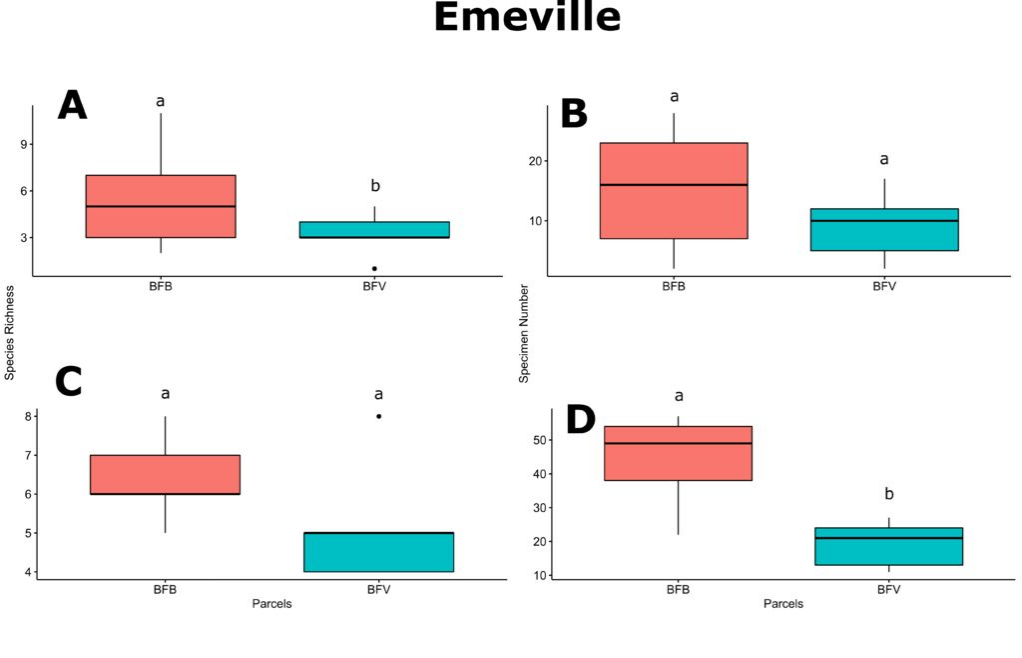
Mean values of species richness and abundance for bee and hoverfly fauna amongst flower strips BFB and BFV (see details given in Table 1). **A.** Bee richness; **B.** Bee abundance; **C.** Hoverfly richness; **D.** Hoverfly abundance. Letters above the boxplots represent Student t-test comparisons.

For Froidefontaine farmstead, bee richness in VER was significantly higher than in GC (*p*-value < 0.05; Fig. 3A) and bee abundance in PAT was significantly higher than in GC, VER and ZH (*p*-values < 0.05; Fig. 3B). Hoverfly diversity in ZH was significantly higher than in VER (*p*-value < 0.05; Fig. 3C), while hoverfly abundance was homogenous amongst the Froidefontaine parcels (Fig. 3D). For Emeville farmstead, bee and hoverfly richness and bee abundance did not vary amongst parcels (Fig. 4A, B and C), while DIK parcel exhibited significantly greater hoverfly abundance than EPI, FRE and PAV parcels (*p*-values < 0.05; Fig. 4D). Only for bee richness and hoverfly abundance, the pollinator flower patch BFB showed significantly higher mean values than the feeder flower patch BFV (*p*-values;Fig. 5A and D).

## Discussion

### Polylectic bee species

In our study, we identified 101 different bee species, corresponding to almost one quarter of the Belgian bee fauna (Drossart et al. 2019). Depicting 57.32% of the total bee collected material, the top-five bee species in both farms were *Andrena cineraria* (19.59%), *Apis mellifera* (12.94%), *A. haemorrhoa* (10.71%), *A. flavipes* (7.25%) and *Lasioglossum pauxillum* (6.83%).

Both farms presented suitable habitats to these polylectic species, including open wooded spaces, fallow land or lawns. The abundance of *Taraxacum* spp. (Asteraceae), *Salix* spp. (Salicaceae), *Craetegus* spp. (Rosaceae) and fruit trees could explain the dominance of *A. cineraria*, *A. haemorrhoa* and *A. flavipes* populations. Moreover, they usually nest in south-exposed sites, in bare soils or in areas with sparse and short vegetation (Falk 2015). The other common polylectic bees were mainly ground-nesting species belonging to *Andrena* and *Lasioglossum* genera, such as *A. nitida*, *A. gravida*, *L. calceatum* or *L. lativentre* (Table 2).

**Figure 6.**
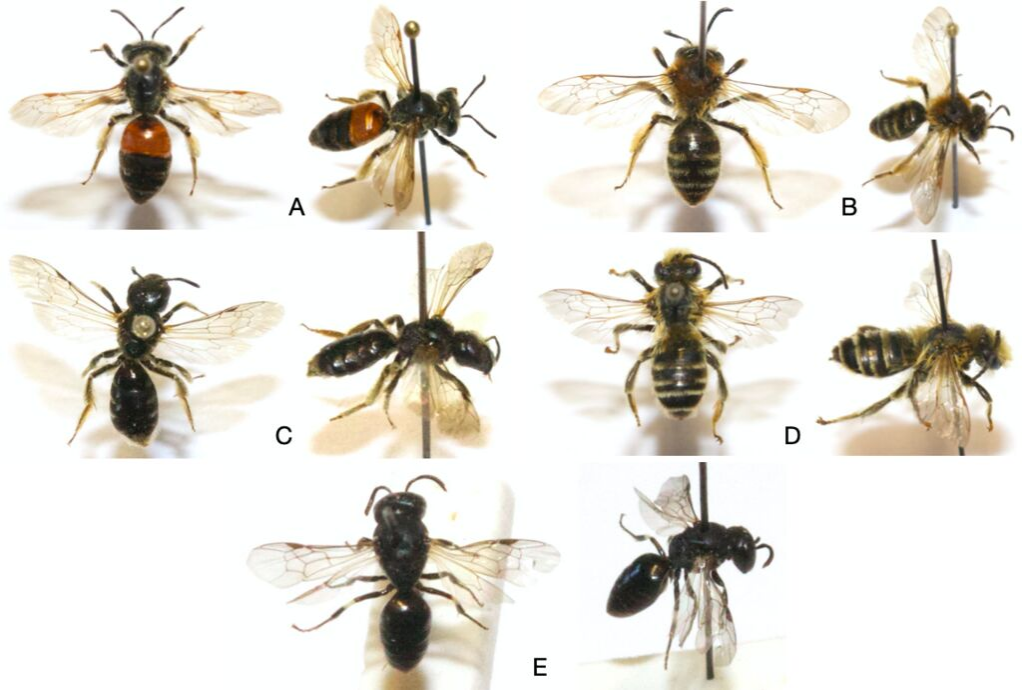
Dorsal and lateral side of some rare bees observed within the farmsteads. **A.** *Andrena schencki* Morawitz 1866; **B.** *Andrena fulvata* (Müller 1766); **C.** *Halictus maculatus* Smith 1848; **D.** *Melitta leporina* (Panzer 1799); **E.** *Hylaeus brevicornis* Nylander 1852.

Uncommon polylectic bee species were also collected. For example, *Andrena trimmerana* and *Halictus maculatus* (Fig. 6C) are rarely observed in the Condroz Region and more largely in Belgium. *H. maculatus* is a little more common in Wallonia and this species is considered as "vulnerable" in Belgium, but "least concern" in Europe (Drossart et al. 2019, Nieto et al. 2014). Moreover, this species forages on *Achillea millefolium* (Asteraceae), *Centaurea* spp. (Asteraceae) or *Daucus carota* (Apiaceae) (Pauly 2019), which were naturally present or cultivated in both farms. In 2019, specimens of *A. trimmerana* were collected only in the Froidefontaine farmstead, where *Rubus* spp. (Rosaceae), orchards, umbellifers or *Cirsium* spp. (Asteraceae) were flowering. Two specimens of *Colletes cunicularius* were sampled from both farms. This species is specialised on *Salix* spp. (Salicaceae) or *Prunus cerasus* L., (Rosaceae) (Falk 2015). While *Lasioglossum leucopus* was observed in both farms - probably because of the presence of several of its preferred host plants, *Ranunculus* spp. (Ranunculaceae), *Taraxacum* spp. (Asteraceae) and *D. carota* - this species is considered as "near threatened" according to the IUCN Red List Criteria in Belgium (Drossart et al. 2019, Pauly 2019).

Rarer species were observed within the farmsteads. Collected in the orchard of Froidefontaine, *Andrena schencki* (Fig. 6A) had not been observed south of the Sambre and Meuse Furrow for more than 30 years (Rasmont and Haubruge 2002). *Andrena semilaevis*, a very rare species since 1990 in Belgium (Rasmont and Haubruge 2002), was captured in the orchard of Emeville. This polylectic species is mostly observed on the umbellifers (Falk 2015). Forty-six specimens (1.12% of total sampling) of *Andrena fulvata* (Fig. 6B) were collected in 2019 in all habitats of both farms, while only one observation was encoded in Atlas Hymenoptera repository for Belgium (Rasmont and Haubruge 2002). That probably means a recent installation of the population on the study sites. However, misidentification due to their morphological resemblance to *A. angustior* could bias its Belgian rarity (T.J. Wood, personal communication). This species nests in calcareous soils and forages principally on Asteraceae flowers, such as *Taraxacum* spp. (Falk 2015).

The high diversity of wild bees in the two farms could be linked to the presence of semi-natural habitats around the parcels. Indeed, the implantation of hedgerows, flower strips or shrubby strips between the habitats of both farms provides sufficient floral resources during the foraging activity period of polylectic species (Albrecht et al. 2020).

### Oligolectic bee species

Thirteen bee species were characterised as oligolectic (Drossart et al. 2019), which represented 24 specimens (Table 2).

Two common species, *A. praecox* and *A. vaga* and two uncommon species, *A. apicata* and *A. mitis*, were collected in different parts of both farms (Table 2). In Belgium, they are considered as *Salix* spp. specialists. Moreover, these last two species had never been observed in Condroz before and not since 1950 in the south of Wallonia. *A. humilis* is a specialist of Asteraceae plant species, such as *Tragopogon dubius* Scopoli 1772, *Hieracium pilosella* Vaillant 1754 (Scheuchl 2002) or *Cichorium* spp. and *A. labialis* is a specialist of leguminous plants (Fabaceae) (Rasmont and Haubruge 2002).

A single specimen of Melittidae family, *Melitta leporina* (Fig. 6D), was sampled. The female is particurlarly related to the flowers of *M. sativa* and *T. pratense* species (Fabaceae) (Dellicour and Michez 2010), which were abundantly present around the wetland of Froidefontaine Farm. One species of Colletidae family, *Colletes daviesanus*, forages pollen entirely from composite flowers such as tansy, mayweeds or oxeye daisy (Asteraceae) (Falk 2015).

In Froidefontaine habitats, we also sampled a few specimens of *Chelostoma rapunculi*, *Eucera longicornis*, *Hylaeus signatus*, *Megachile ericertorum* and *Osmia leaiana*, probably because their preferred flowers were partially present:*Trifolium* sp., *Medicago* sp., *Cirsium* sp., *Rubus* sp., *Centaurea* sp. and *Stachys sylvatica* L. 1753.

### Cuckoo bee species

We only collected two specimens of cuckoo bumble bees (subgenus Psithyrus Lepeletier), *Bombus campestris* and *B. vestalis*, in Froidefontaine wetland and in Froidefontaine orchard (Table 2), respectively. They are considered rare species (Lhomme and Hines 2018) and their presence could be explained by the relative predominance, in the *Bombus* genus, of their associated host species: *B. pascuorum* and *B. terrestris* (Table 2).

Concerning the nomad bees (*Nomada* spp.), we identified 15 species representing 4.6% of the collected material. They especially parasitise *Andrena* spp. and their relative abundance is dependent on the proportion of their host bee species (Sheffield et al. 2013). Most of their host species were collected throughout the two years of experiment. For example, we found, in a small proportion, *Nomada flavoguttata* and *N. leucophthalma*, which are linked to *Micrandrena* spp. Ashmead 1899 (*Andrena semilaevis*, *A. subopaca*...) and *A. apicata*, respectively. On the contrary, *N. goodeniana* and *N. ruficornis* were largely present due to the strong dominance of *A. cineraria* and *A. haemorrhoa* (Rasmont and Haubruge 2002).

All collected *Sphecodes* spp. are generalist cleptoparasites, except for *S. gibbus* that parasitises the nests of *Halictus* species, such as *H. maculatus* and *H. rubicundus*. Their relative abundance followed also the abundance of their host species: the most collected *S. epphipius* is the cuckoo bee of the most collected halictid bee, *Lasioglossum pauxillum* (Pauly 2019).

### Hoverfly species

Within both farmsteads, *Sphaerophoria scripta* was, by far, the most abundant hoverfly species, followed by *Eristalis tenax* and *Episyrphus balteatus*, corresponding together to almost three quarters of the total number of collected specimens (Table 2). These species are the most common syrphids encountered in Central Europe (Alhmedi et al. 2010, Francuski et al. 2013, Nengel and Drescher 1990). Aphidophagous larvae of *S. scripta* and *E. balteatus* are important for pest control in agricultural systems, while *E. tenax* larvae recycle the organic matter in wet manures, muds or ponds (Sommaggio 1999). We also emphasised the presence of *Melanostoma mellinum*, which occured in almost each habitat and particularly in flower strips. Adults *M. mellinum* are specialised in the floral visitation of anemophilous plants (Van der Groot and Grabandt 1970).

**Figure 7.**
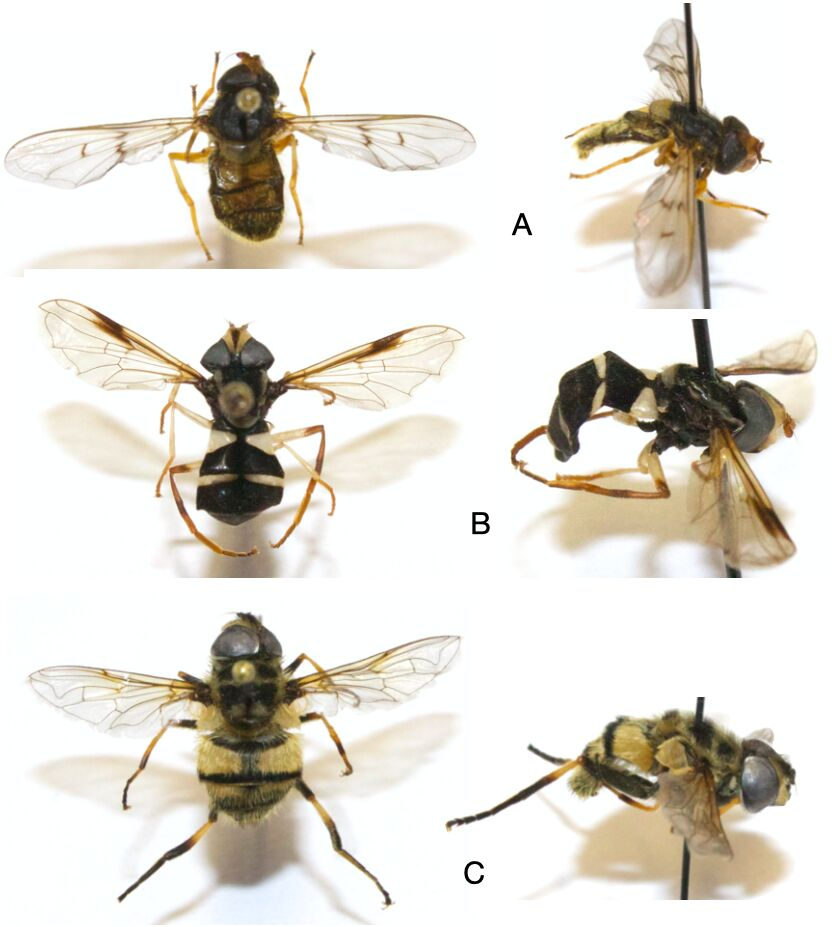
Dorsal and lateral side of some rare hoverfly species observed within the farmsteads. **A.** *Ferdinandea cuprea* (Scopoli 1763); **B.** *Xanthogramma pedissequum* (Harris 1776); **C.** *Myathropa florea* (L.).

Beside these ubiquitous species, rarer species were found in only a few habitats: *Xanthogramma pedissequum*, *Myathropa florea* and *Ferdinandea cuprea* (Fig. 7). Unlike *S. scripta* and *E. tenax*, these species do not migrate. The larvae of *X. pedissequum* feed on aphids reared on the anthills of some *Lasius* sp. Fabricius 1804 (Hymenoptera: Formicidae) (Speight 2020). The species *M. florea* and *F. cuprea* present a microphagous larval stage. In intensified agricultural landscapes, it is conceivable that the environmental requirements of such species are scarcely fulfilled. Notably, microphagous species appear to be particularly sensitive to pesticides (Schweiger et al. 2007). On the contrary, agricultural landscapes of Froidefontaine and Emeville Farms are suitable for these specialist species, because they include semi-natural ecosystems and organic orchards where cattle or sheep are grazing. We also identified two specimens of *Platycheirus immarginatus* that are specialist foragers on *Bolboschoenus maritimus* (L.) (Table 2) (Speight 2020). We did not find this plant species in Froidefontaine farmstead, meaning that *P. immarginatus* might forage on other plant species.

Continuous sampling represented only 4.33% of the total hoverfly specimens. However, it allowed us to reveal two more hoverfly species, in Emeville flower strips: *Xylota sylvarum* and *X. segnis*, whose larvae are saproxylic and live close to roots and dead wood (Speight et al. 2000).

### Impact of agroecological practices on wild bees and hoverflies communities at the farm scale

By in-depth sampling, we documented new occurrences of almost 1/4 of Belgian bee fauna in two farms in ecological transition. For the historical region of the Municipality of Havelange, we have almost quintupled the richness of wild bees community despite high quality monitoring of these populations in Belgium (Drossart et al. 2019). There are few studies of this type in a close environment and with comparable methodology. Therefore, comparing our results with other studies seems to be of little relevance. This study leads us to consider that, on small areas undergoing ecological transition, an important richness of pollinators is easily reached. Moreover, it is possible that the conducted survey underestimates the real diversity per plot, even if the pattern of dominance rarity should be maintained. We also lack data at the end of the season, especially for late summer bees, such as *Colletes hederae* (Schmidt and Westrich 1993). For hoverflies, we still lack inventory data on the scale of the Belgian territory (Frank Van de Meutter, pers. comm.).

**Figure 8.**
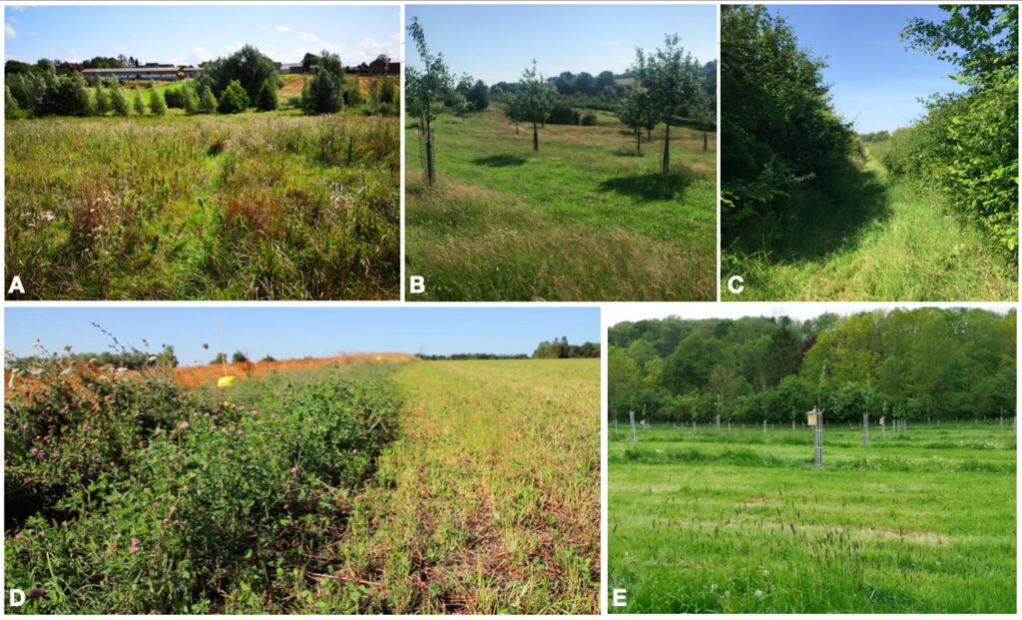
Some field pictures in each farm. **A.** Froidefontaine wetland (ZH); **B.** Froidefontaine orchard (VER); **C.** Double hedgerow between Froidefontaine cultivated parcel (GC) and pasture (PAT); **D.** Emeville flower strip between FRE and EPI parcels (photo credit : I. Van Dorpe); **E.** Emeville orchard (PAV).

The practices on and around the studied farms seemed favourable to pollinators (Fig. 8) and especially to the polylectic species. In Froidefontaine Farm, the land tenure showed strong impact on bee richness and abundance by an alternation of floral bee-feeding parcels, like the Froidefontaine pasture (PAT; Fig. 3B) and bee-nesting parcels, like the Froidefontaine orchard (VER; Fig. 3A & Fig. 8B). On the one hand, late mowing permits the keeping of abundant floral resources throughout the bee activity period (Meyer et al. 2017) and, on the other hand, sheep grazing permits theconservation of some bare soil sites that favour ground-nesting bees (Cane 1991). Landscape micro-habitats, such as ponds, hedgerows or groves, are important to the survival of many pollinator species, especially by providing habitats for hoverfly larvae (Sommaggio 1999). The wetland of Froidefontaine (ZH) (Fig. 8A) harboured higher hoverfly diversity than the other parcels (Fig. 3C), with species like *S. scripta*, *Cheilosia* sp. and *E. tenax*, whose larvae have different diets (i.e. aphidophagous, phytophagous and microphagous, respectively) (Sommaggio 1999, Speight 2020). The cultivated parcel of Froidefontaine (GC) (Table 2) and the pea crop of Emeville (DIK) (Fig. 4D) showed high abundances of aphidophagous hoverflies, likely caused by the high prevalence of aphids on crops. The flower strips separating the parcels of Emeville Farm consisted of a floral mix especially designed to fill the ecological requirements of bees and hoverflies (Fig. 8D). The floral composition of these flower strips attracted more hoverfly specimens than bees, which were mainly represented by *A. mellifera* (Table 2). Moreover, they were combined with belatedly-mowed hedges that support floral resources for pollinators throughout their activity season. Similarly, the hedgerows bordering the parcels of Froidefontaine (Fig. 8C), coupled with ecological crop management practices (i.e. no-till, no chemical inputs...), promoted the establishment of wild bee populations (Albrecht et al. 2020). Indeed, hedgerows and other semi-natural habitats usually represent superior floral richness and abundance compared to intensive agricultural land use (Hannon and Sisk 2009).

According to the Belgian Red List of bees (Drossart et al. 2019), we have collected several species indexed in threatened categories from diverse habitats of both farms, especially in the orchard and in the wetland of Froidefontaine. These species were represented by one specimen of *A. schencki*, one specimen of *B. campestris*, one specimen of *E. longicornis* and nine specimens of *H. maculatus*. We also mitigated the data deficiency in Belgium for a few records of bee species, such as *A. semilaevis*, *A. trimmerana* and *Hylaeus brevicornis* (Fig. 6E). Taxonomically recent recognition, split from species complex and morphological similarity with widespread taxa or less studied genera (e.g. *Hylaeus* sp.) reflect current taxonomic impediments for 9.4% of the Belgian bee richness (Drossart et al. 2019).

Pollinator composition of each farmstead harboured both common and rare species, which indicates that on-farm diversification and organic practices may be an important refuge for rare, Red-Listed or oligolectic pollinator species (Guzman et al. 2019). Restoring or incorporating diverse habitats in agro-ecosystems is therefore a long-term solution for the conservation of pollinating species (Saint Clair et al. 2020).

## Supplementary Material

FA2F290F-667A-5394-B421-364EC9D03A78 10.3897/BDJ.9.e60665.suppl1 Supplementary material 1 Havelange bee historical data Data type occurences File: oo_472961.xlsm https://binary.pensoft.net/file/472961 Grégoire Noël

## Figures and Tables

**Table 1.** Habitats description of the sampled parcels and flower strips.

Parcel Name	Parcel Code	Farmstead	Sampling Year	Parcel description
Pavillon	PAV	Emeville	2019	Pastures combined with apple orchard. Flowering fields under young apple trees (many rustic varieties). There are dandelions, shamrocks, meadow cardamine etc. This parcel is mainly surrounded by woods and hedges. A herd of Angus was grazing most of the time, from May.
Frere	FRE	Emeville	2019	Mainly alfalfa, some other fabaceae (red and white clovers). There are dandelions and speedwells at the start of the season. A hedge borders the parcel to the East. A flowery strip runs on the South face (BF 2; Fig. 2B). Harvested during the month of June and after recovery in mid-July.
Epicurien	EPI	Emeville	2019	Divided parcel along the East to the West, composed equally of small and large spelts. Hedgerows border the parcel to the East face.
Dikkekip	DIK	Emeville	2019	The parcel is at the bottom of the slope. Left without plant cover until May, when pea crop was sown. There are some rumex and a lot of chamomile too.
Flower strips	BF	Emeville	2019	Composed of a mix of cover crops and flower crops. See the site description for more details.
Crops	GC	Froidefontaine	2018-2019	Vegetable crops occupy a third of the surface of the cultivated parcel.
Pasture	PAT	Froidefontaine	2018-2019	A hay meadow composed of Poaceae, clovers, dandelions etc. Bordered by hedgerows, except to the South face (sheep fence).
Orchard	VER	Froidefontaine	2018-2019	Flowering fields under young apple trees (many rustic varieties). This parcel is grazed by sheep in April and May. The parcel is bordered by hedges, except to its North face (sheep fence).
Wetland	ZH	Froidefontaine	2018-2019	The vegetation is mainly composed of plants from wetlands: buttercups, nettles, thistles, cradles etc. The meadow is bordered by a brook to the South and a hedge to the North.

**Table 2.** Abundance of each pollinator species according to the habitat of its collection. The habitat details are given in Table 1.

	BF	DIK	EPI	FRE	GC	PAT	PAV	VER	ZH	Total (%)
Bee	285	256	277	244	439	1145	349	685	623	4303 (100)
*Andrena angustior*		1	2		13	17	1	10	13	57 (1.32)
*Andrena apicata*		1								1 (0.02)
*Andrena bicolor*		1		1	7	1		4	10	24 (0.56)
*Andrena carantonica*				1		5		4	3	13 (0.3)
*Andrena chrysosceles*		2	5	5	1	2	6	1	4	26 (0.6)
*Andrena cineraria*		9	25	42	117	409	42	90	109	843 (19.59)
*Andrena dorsata*		6	4	7		6	6	12	5	46 (1.07)
*Andrena flavipes*	4	15	16	10	57	54	20	73	63	312 (7.25)
*Andrena fulva*		1	1	3	4	7	7	8	1	32 (0.74)
*Andrena fulvata*		6	10	1	7	3	6	9	6	48 (1.12)
*Andrena gravida*		2	3	3	8	48	4	23	15	106 (2.46)
*Andrena haemorrhoa*	1	7	5	19	32	145	24	121	107	461 (10.71)
*Andrena humilis*			1		2	1	1		2	7 (0.16)
*Andrena labialis*	2									2 (0.05)
*Andrena labiata*			1					2		3 (0.07)
*Andrena minutula*	1	3	2	1	2	1	2	3		15 (0.35)
*Andrena mitis*		2								2 (0.05)
*Andrena nigroaenea*		5	3	2	13	9	3	6	13	54 (1.25)
*Andrena nitida*		4	10	8	11	60	24	40	20	177 (4.11)
*Andrena ovatula*					3			1		4 (0.09)
*Andrena praecox*									3	3 (0.07)
*Andrena schencki*								1		1 (0.02)
*Andrena semilaevis*							1			1 (0.02)
*Andrena subopaca*	2		1			1			1	5 (0.12)
*Andrena trimmerana*					1	1				2 (0.05)
*Andrena vaga*		2	2	3		4	3	1		15 (0.35)
*Andrena wilkella*	8							6	1	15 (0.35)
*Apis mellifera*	114	32	33	35	57	63	128	54	41	557 (12.94)
*Bombus campestris*									1	1 (0.02)
*Bombus hortorum*	1		2	2		4			1	10 (0.23)
*Bombus hypnorum*						3			3	6 (0.14)
*Bombus lapidarius*	35	1	4	5	4	73	7	29	50	208 (4.83)
*Bombus pascuorum*	58		1	13	7	26	1	7	20	133 (3.09)
*Bombus pratorum*		1		2	2	9	1	3	4	22 (0.51)
*Bombus terrestris*	35	2	2	12	19	17	8	12	18	125 (2.9)
*Bombus vestalis*								1		1 (0.02)
*Chelostoma rapunculi*						1				1 (0.02)
*Colletes cunicularius*				1				1		2 (0.05)
*Colletes daviesanus*					1					1 (0.02)
*Eucera longicornis*								1		1 (0.02)
*Halictus maculatus*			1		1	1		3	3	9 (0.21)
*Halictus rubicundus*						2	1		2	5 (0.12)
*Halictus scabiosae*								2	2	4 (0.09)
*Hylaeus brevicornis*								2		2 (0.05)
*Hylaeus communis*				1			1			2 (0.05)
*Hylaeus hyalinatus*								1		1 (0.02)
*Hylaeus signatus*	1									1 (0.02)
*Lasioglossum calceatum*		38	43	14	9	29	11	24	16	184 (4.28)
*Lasioglossum fulvicorne*		2					2			4 (0.09)
*Lasioglossum laticeps*		1	4	1	4	1	1	2	1	15 (0.35)
*Lasioglossum lativentre*	5	1	16		2	17	4	28	1	74 (1.72)
*Lasioglossum leucopus*		3	1		1					5 (0.12)
*Lasioglossum leucozonium*		4	3	1	1	4	1	5	2	21 (0.49)
*Lasioglossum malachurum*			1		1		1		1	4 (0.09)
*Lasioglossum morio*		8	1			3	1	2	2	17 (0.4)
*Lasioglossum pauxillum*	6	93	62	41	19	24	13	16	20	294 (6.83)
*Lasioglossum punctatissimum*			1	1			1	1	1	5 (0.12)
*Lasioglossum sexstrigatum*			1							1 (0.02)
*Lasioglossum* sp.					1		1	2		4 (0.09)
*Lasioglossum villosulum*		1	1		4	2	1	3		12 (0.28)
*Lasioglossum zonulum*		1	1	3	4	4	1	2	4	20 (0.46)
*Megachile ericetorum*	1								1	2 (0.05)
*Megachile willughbiella*	1							1		2 (0.05)
*Melitta tricincta*								1		1 (0.02)
*Nomada bifasciata*						2	1	2	1	6 (0.14)
*Nomada fabriciana*						1		1	1	3 (0.07)
*Nomada flava*			1			2		2	2	7 (0.16)
*Nomada flavoguttata*						1		1	3	5 (0.12)
*Nomada fucata*	1		2		4	7	5	11	1	31 (0.72)
*Nomada fulvicornis*			1	2			1			4 (0.09)
*Nomada goodeniana*			2		5	24	2	13	7	53 (1.23)
*Nomada lathburiana*					1	9	1	2	2	15 (0.35)
*Nomada leucophthalma*						2		1	1	4 (0.09)
*Nomada marshamella*									2	2 (0.05)
*Nomada panzeri*						2			3	5 (0.12)
*Nomada ruficornis*			1		1	21		13	17	53 (1.23)
*Nomada signata*						2		1	1	4 (0.09)
*Nomada succincta*						1				1 (0.02)
*Nomada zonata*						1	1	2	1	5 (0.12)
*Osmia bicolor*									1	1 (0.02)
*Osmia bicornis*					3	6	1	2	5	17 (0.4)
*Osmia cornuta*						1		2		3 (0.07)
*Osmia leaiana*							1	1		2 (0.05)
*Osmia leucomelana*	1				1			2		4 (0.09)
*Osmia tridentata*	1									1 (0.02)
*Seladonia tumulorum*	7	1		3	4	3		6	2	26 (0.6)
*Sphecodes ephippius*			1	1	3	3		3	1	12 (0.28)
*Sphecodes ferruginatus*							1			1 (0.02)
*Sphecodes gibbus*									1	1 (0.02)
*Sphecodes monilicornis*								1	1	2 (0.05)
*Sphecodes puncticeps*					1					1 (0.02)
*Sphecodes* sp.					1			1		2 (0.05)
Hoverfly	907	228	26	91	266	91	86	72	231	1998 (100)
*Cheilosia* sp.	2				1	15	1	1	55	75 (3.75)
*Episyrphus balteatus*	124	10	1	36	6	3	5	10	14	209 (10.46)
*Eristalis arbustorum*	60	10	3	1	5	2	2	5	15	103 (5.16)
*Eristalis nemorum*	3									3 (0.15)
*Eristalis pertinax*								1	5	6 (0.3)
*Eristalis sepulchralis*	1									1 (0.05)
*Eristalis similis*								1		1 (0.05)
*Eristalis tenax*	186	13	4	8	37	24	43	4	23	342 (17.12)
*Eupeodes luniger*	6	9	1	1	3	1	1	1	2	25 (1.25)
*Ferdinandea cuprea*							2			2 (0.1)
*Helophilus trivittatus*						1			2	3 (0.15)
*Melanostoma mellinum*	53	17		13	13	1	3	1	1	102 (5.1)
*Metasyrphus corollae*	7	15		3	1	1	1	2		30 (1.5)
*Metasyrphus latifasciatus*						2			4	6 (0.3)
*Myathropa florea*				1					3	4 (0.2)
*Platycheirus albimanus*	1	1			1					3 (0.15)
*Platycheirus clypeatus*									1	1 (0.05)
*Platycheirus immarginatus*						1		1		2 (0.1)
*Platycheirus peltatus*						3	1		2	6 (0.3)
*Platycheirus scambus*					2					2 (0.1)
*Rhingia campestris*						1				1 (0.05)
*Scaeva pyrastri*	19	5		3	4	3	1		3	38 (1.9)
*Sphaerophoria scripta*	401	148	13	23	174	29	17	40	84	929 (46.5)
*Syritta pipiens*	37		1		13			1	4	56 (2.8)
*Syrphus ribesii*	3		3	1	3	3	9	1	10	33 (1.65)
*Syrphus vitripennis*	1				3	1		1	2	8 (0.4)
*Volucella bombylans*								1		1 (0.05)
*Volucella pellucens*								1		1 (0.05)
*Xanthogramma pedissequum*				1					1	2 (0.1)
*Xylota segnis*	2									2 (0.1)
*Xylota sylvarum*	1									1 (0.05)
Total of specimens	1192	484	303	335	705	1236	435	757	854	6301
